# Farm family effects of adopting improved and hybrid sorghum seed in the Sudan Savanna of West Africa

**DOI:** 10.1016/j.foodpol.2018.01.001

**Published:** 2018-01

**Authors:** Melinda Smale, Amidou Assima, Alpha Kergna, Véronique Thériault, Eva Weltzien

**Affiliations:** a Department of Agriculture, Food and Resource Economics, Michigan State University, East Lansing, MI, USA; b Economie de la Filière, Institut d’Economie Rural, Bamako, Mali; c Honorary Fellow, Department of Agronomy, University of Wisconsin, Madison, WI, USA

**Keywords:** Sorghum, Improved varieties, Mali, Multivalued treatment, Farm family

## Abstract

Uptake of improved sorghum varieties in the Sudan Savanna of West Africa has been limited, despite the economic importance of the crop and long-term investments in sorghum improvement. One reason why is that attaining substantial yield advantages has been difficult in this harsh, heterogeneous growing environment. Release in Mali of the first sorghum hybrids in Sub-Saharan Africa that have been developed primarily from local germplasm has the potential to change this situation. Utilizing plot data collected in Mali, we explain the adoption of improved seed with an ordered logit model and apply a multivalued treatment effects model to measure impacts on farm families, differentiating between improved varieties and hybrids. Since farm families both consume and sell their sorghum, we consider effects on consumption patterns as well as productivity. Status within the household, conferred by gender combined with marital status, generation, and education, is strongly related to the improvement status of sorghum seed planted in these extended family households. Effects of hybrid use on yields are large, widening the range of food items consumed, reducing the share of sorghum in food purchases, and contributing to a greater share of the sorghum harvest sold. Use of improved seed appears to be associated with a shift toward consumption of other cereals, and also to greater sales shares. Findings support on-farm research concerning yield advantages, also suggesting that the use of well-adapted sorghum hybrids could contribute to diet diversification and the crop’s commercialization by smallholders.

## Highlights

• The first sorghum hybrids based on local germplasm in West Africa yield well on farms.
• Intrahousehold status on extended family farms affects adoption of improved seed.
• Adoption raises yield, reduces the harvest share consumed and also purchased.
• Adoption increases the harvest share sold and dietary diversity.

## 1 Introduction

Sorghum is grown in harsh environments where other crops grow poorly, by farmers who are among the world’s poorest (Dalton and Zereyesus, 2013). Along with millet and groundnuts, sorghum “defines” the semi-arid tropics of West Africa (Ndjeunga et al., 2015). Since the devastating droughts of the 1970–80s, national and international research institutes have invested to improve sorghum productivity in this region (Matlon, 1985, Matlon, 1990). Globally, when combined with other farming practices, the diffusion of well-adapted, improved seed has enhanced the productivity of major food crops, including sorghum (Evenson and Gollin, 2003). Yet, area shares planted to improved sorghum varieties are estimated at only 3% in Burkina Faso, 15% in Niger, and 20% in Nigeria (Ndjeunga et al., 2015). In Mali, our country of study, estimates range from 13% to around 30%, depending on the measurement approach, geographical coverage, and time period (Kelly et al., 2015).

From a plant breeding perspective, one explanation for low adoption rates is that under farmers’ conditions, achieving sizeable yield gains with improved sorghum varieties has posed particular challenges. In the Sudan Savanna of West Africa, yield advantages have not attained 30% over farmers’ own best cultivars until the release in 2010 of the first sorghum hybrids based largely on Guinea-race germplasm that originated in this region (Rattunde et al., 2013). Earlier research had demonstrated the potential of hybrid sorghum seed to yield well under controlled conditions in Niger and Kenya, but with introduced germplasm (e.g., House et al., 1997, Haussmann et al., 1998).

From a policy perspective, numerous structural constraints have impeded adoption, including a slow transition from entirely state-managed seed supply channels (e.g., Haggblade et al., 2015, Coulibaly et al., 2014). Most smallholder farmers in the drylands have few resources other than seed, their labor, and their family lands to produce the cereal harvests they need to survive. From the perspective of a Malian farmer, one might argue that no seed value chain has existed for sorghum (Siart, 2008). Most of Mali’s numerous sorghum growers remain largely outside the formal structures of extension and program services, termed “encadrement” (Thériault et al., 2015). By comparison, cash crops such as rice or cotton (and its primary rotation crop, maize) have vertically-integrated value chains that provide a range of services via registered cooperatives.

Others argue that farmers in this region avoid paying cash for seed--and understandably so, if the advantages of using improved seed are dubious. Sorghum is a traditional food staple in Mali and the West African Savanna is one of the centers of the crop’s domestication. The fundamental role that sorghum seed plays in the well-being of smallholder farmers is reflected in rural cultural norms. These include a customary reverence for the seed of local varieties, a perspective that all farmers have a right to seed, and a preference for giving or sharing seed rather than exchanging or purchasing seed with cash (Siart, 2008, Smale et al., 2010). The sorghum seed system has remained ‘farmer-centered’ (Haggblade et al., 2015), with farmers themselves diffusing much of the local and improved seed plant each season through associations, social networks and other relationships based on trust. There is some evidence that preferences regarding seed acquisition may be evolving, however. For example, our census of sorghum varieties grown by 2430 growers in the Sudan Savanna indicated that 38% of seed of improved varieties and 67% of seed of hybrids was initially obtained through a cash purchase as compared to gifts or exchanges.

In this paper, we explain adoption and measure the impacts of improved sorghum seed on farm families at an initial point in the diffusion process for hybrids in the Sudan Savanna of West Africa, where they were initially introduced in Mali. We make several contributions to the literature on this topic. First, we measure the farm family impacts of the first sorghum hybrids developed and released in Sub-Saharan Africa that were based largely on local germplasm. In addition, these were bred through an on-farm, participatory process. We employ data generated by statistical sample drawn from 58 villages in Mali and we are able to differentiate hybrids from other improved varieties based on expert identification.

Second, instead of grouping improved varieties with hybrids, we differentiate the impacts of each seed type by applying a less frequently used, multivalued treatment effects approach. Described by Wooldridge, 2010, Cattaneo, 2010, and Cattaneo et al. (2013), we have found only one published example so far of its application in agriculture (Esposti, 2017). Many treatment applications with observational data in agricultural development pertain to binary assignment, which is typically addressed with propensity score matching—an approach that has not been fully extended to multivalued treatments. We test three multivalued effect estimators with regression methods. Regression (RA) serves as a baseline estimator. We also apply the augmented, inverse-probability weighted (AIPW) estimator and the inverse-probability weighted, regression adjustment (IPWRA) estimator, both of which are described as “doubly robust.”

Third, considering that like most of the smallholders in the Sudan Savanna of West Africa, Malian farm families both sell and consume their crop, we explore effects of improved seed use not only on plot-level yield but also household dietary diversity and the share of sorghum in consumption and sales. Finally, reflecting the social organization of production among sorghum growers in this region, we include the gender, generation and education of the plot manager in addition to plot, household and market characteristics among our control variables. These covariates also enable us to capture intrinsic, unobserved management factors related to the status of the plot manager within the household.

## 2 Context

Three aspects of sorghum production in the Sudan Savanna of West Africa are fundamental to understanding the process and potential of seed-based technical change: the nature of sorghum genetic resources in the region, including the history of sorghum improvement; the importance of the crop in dryland farming; and the social organization of sorghum production.

Farmers in Sub-Saharan African grow several morphological forms or “races” of sorghum. These include caudatum (originating in eastern Africa), durra (grown principally in the Horn of Africa, and with residual moisture), kafir (cultivated in southern Africa), and Sorghum bicolor, which is broadly distributed. The West African Savanna, where parts of the Guinea race of sorghum originated and still dominates (Olsen, 2012), produces most of the sorghum in Sub-Saharan Africa. The Guinea race of sorghum is uniquely adapted to growing conditions in the West African Savanna. Photo-period sensitivity enables its plants to adjust to the length of the growing seasons, which is important for farmers when the beginning of the rainy season is unpredictable. The lax panicles and open glumes of this morphological form reduce grain damage from birds, insects and grain mold (Rattunde et al., 2013).

Sorghum and millet continue to serve as the cereals base of the drylands agricultural economy in the Sudan Savanna of West Africa—destined primarily for consumption by the farming families who grow them, but also sold for cash as needed. Recognizing the importance of sorghum as a food staple in more arid zones, the governments of Mali, Burkina Faso and Niger, in particular, have long pursued a goal of raising sorghum productivity. During the Sahelian droughts of the 1970–1980s, national and international research systems accelerated efforts to enhance sorghum yields, also introducing exotic germplasm from outside national borders (Matlon, 1985, Matlon, 1990, Ndjeunga et al., 2015).

Nonetheless, growth rates of national yields have not been as impressive as might be hoped. Yields reported by FAOSTAT (2016) for Mali show an average growth rate of 0.49% from 1961 to 2013. From 1980 to 2013, which corresponds to an active sorghum improvement program, the average growth rate in sorghum yields was 2.3%. This growth is quite modest, especially when compared with the 7.6% average growth rate in rice yields over the same time period. National average yields have rarely exceeded 1 t per ha.

In Mali, sorghum is extensively cultivated on degraded soils with low fertility and little to no chemical fertilizer. Early breeding approaches focused on “purification” of superior farmers’ varieties and the introduction of exotic germplasm, but this latter group often lacked resistance to insects and grain mold and farmers’ varieties were often preferred for their grain quality (Yapi et al., 1990). In general, achieving more than marginal yield changes has been difficult without hybrid vigor. Since 2000, the national sorghum improvement program has pursued two additional directions. The first is a participatory approach to sorghum improvement, based on a network of multi-locational, farmer field trials managed by farmer associations. The second is the development of the first hybrid seed based primarily on germplasm of the local Guinea race of sorghum. Summarizing the results of trials conducted by smallholder farmers in the Sudan Savanna, Rattunde et al. (2013) reported yield advantages of individual hybrids of 17–47% over the local check, with the top three hybrids averaging 30%. Such yield advantages had not been previously achieved with improved varieties in this region. On-farm trials represented a broad range of growing conditions, including entries grown with and without fertilizer.

Aside from these sorghum hybrids, no other hybrid sorghum seed based on local germplasm has been released to farmers in Sub-Saharan Africa. A private hybrid was introduced in the irrigated areas of Sudan (Ndjeunga et al., 2015) and other exotic hybrids have been tested in Niger and Kenya (House et al., 1997, Haussmann et al., 1998), The development and release in Mali of hybrid seed based largely on local, Guinea-race sorghum is thus a pilot initiative for the broader region of the West African Savanna and a major step for sorghum improvement in Sub-Saharan Africa.

Dryland cereals in the Sudan Savanna of West Africa are often produced by extended family members who are vertically (unmarried sons, married sons and their families) or horizontally (brothers; multiple wives) related to the head of the family farm enterprise. The head is usually an elder patriarch, or a work leader he designates. The head guides the organization of production on large plots worked collectively with the goal of meeting the staple food needs of the overall extended family. Custodian of the family’s land use rights, he also allocates individual plots to household members who cultivate them privately to meet personal needs. Wives obtain use rights on marriage into the family. Unmarried sons are generally also allocated plots. Status within the extended family household (marked by gender, marital status and generation) conditions rights to land, ability to negotiate for the labor commitment of other household members, and access to family-held equipment and other resources (Thériault et al., 2016, Guirkinger and Platteau, 2014).

## 3 Methods

### 3.1 Data source

The sampling frame is a baseline census of all sorghum-growing households (2430) in 58 villages located in the Cercles of Kati, Dioila, and Koutiala, in the regions of Koulikoro and Sikasso. All variety names reported by farmers and improvement status of varieties (local, improved, hybrid) were identified with the assistance of field technicians and also verified by sorghum breeders working with the national program at the International Crops Research Institute of the Semi-Arid Tropics in Mali. Sikasso and Koulikoro regions have the largest proportions of agricultural land in the Sudan Savanna zone, and are the principal sorghum-producing regions in Mali according to area cultivated and total production. Thus, they are priority target areas for sorghum breeding and especially for hybrid seed development.

Villages surveyed included all those listed as sites where the national research program (Institut d’Economie Rurale-IER) and the International Crops Research Institute for the Semi-Arid Tropics (ICRISAT) have conducted testing activities via a network of farmer associations as early as 2000. Our findings are therefore representative of areas with at least some engagement by the national sorghum program. However, analysis of adoption rates in the baseline census shows variation from 0% to 80% and a distribution that does not differ significantly from normal—enabling us to treat villages as if they had been drawn at random rather than forming a separate control group. Some villages decided not to test varieties; in others, the sorghum program implemented only surveys to familiarize themselves with farmer priorities. Village adoption rates depend on the diffusion strategies pursued by farmer associations and other underlying social networks through which seed is exchanged among farmers, rather than on a formally-managed government program with specified selection criteria.

The enumeration unit in the baseline census, and generally in Mali, is the Entreprise Agricole Familiale (EAF, or family farm enterprise). According to the national agricultural policy act (*Loi d’Orientation Agricole*), the EAF is a production unit composed of several members who are related and who use production factors collectively to generate resources under the supervision of one the members designated as head of household. The head represents the EAF in all civil acts, including representation and participation in government programs. He or she may designate a team leader (*chef de travaux*) to supervise field work and manage the EAF on behalf or to assist the head when he/she has physical or other limitations. In our analytical sample, all but one of the heads is male and all team leaders are male.

For more detailed analysis of adoption and effects of adoption on the well-being of farming households, a sample of EAFs was drawn with simple random sampling using the baseline adoption rate for improved varieties (22%) to calculate sample size. The sample was augmented by five percent to account for possible non-responses, and because of small numbers at this early stage of adoption, all 45 hybrid-growing EAFs were included. The final sample size for adoption and impact analysis is 628 EAFs, with an overall sampling fraction of 25%. Enumerators inventoried all plots operated by each sampled EAF, grouping them by crop and plot management type. One sorghum plot was randomly sampled per management type per EAF. The total sample of sorghum plots analyzed here, including those collectively and individually-managed, is 734. In this analysis, plot is defined by variety; that is, only one sorghum variety is grown per plot. The multi-visit survey was conducted in four rounds from August 2014 through June 2015, with a combination of paper questionnaires and computer-assisted personal interviews, by a team of experienced enumerators employed by the Institut d’Economie Rurale (IER).

### 3.2 Econometric strategy

Our analysis has two components. In the first, we explore the determinants of plot-level variety choice. Estimation of two separate adoption equations (one for improved varieties and one for sorghum) is one feasible strategy, but this strategy would not account for interrelationships in either systematic or random components of the variety choice decision. Bivariate probit would be a modeling option that allows for correlations in the error structure between two separate decisions, but the choice farmers’ make is to grow one type of sorghum variety over another one each plot. Conceptually, we prefer an ordered logit model, differentiating between three types of sorghum varieties: local (0), improved (1), and hybrid (2).

The order, which is sometimes referred to as “improvement status,” recognizes several potentially important differences between the three categories. Many improved varieties grown by farmers in this region are popular older releases, for which the seed may have been reused and shared among farmers. By contrast, sorghum hybrids are new releases. Although on-farm trial evidence demonstrates that sorghum hybrids perform well with and without fertilizer (Rattunde et al., 2013), farmers and extension agents often state that hybrid seed “requires” fertilizer and may manage it differently. In addition, it is recommended that farmers purchase and replace hybrid seed each year, while annual replacement is considered to be less important for improved sorghum varieties as long as good seed storage practices are followed. While we consider that the method of improvement as well as the seed characteristics create an order of choice, we also estimated a multinomial choice model for purposes of comparison.

In the second component of our analysis, we estimate a multivalued treatment effects model. In our context, adoption processes for improved sorghum seed have occurred naturally, with occasional programmatic interventions, over a period of years; treatment assignment is nonrandom because some farmers choose to adopt while others do not. Once introduced into a community by a development project or program, improved sorghum seed, like the seed of local sorghum varieties, has diffused from farmer to farmer based on relationships of trust, including kinship, social networks, formal and informal associations. Thus, we expect that adopters and non-adopters may differ systematically. Sorghum hybrids have been more recently introduced, but through similar processes.

Various methods have been used to address the question of establishing a counterfactual with non-experimental observations, including the class of treatment effect models, most of which involve a single treatment level represented by a binary (0, 1) variable. The case of multivalued treatment effects has been developed by Wooldridge, 2010, Cattaneo, 2010, Cattaneo et al., 2013. Wooldridge (2010: 961-2) presents the example of participation in a training program that occurs at different levels (part-time or full-time), or in different forms. Cattaneo (2010) develops a more general theory for semiparametric estimators and applies it to estimate quantile treatment effects.

Propensity score matching methods have not been fully developed for more than a single value of treatment. As in the binary case, multivalued treatment effects can be estimated by applying regression approaches. Wooldridge (2010; 962-963) recommends RA as a baseline and a simple extension of the binary case to the multivalued case, also suggesting inverse-probability weighting using a multinomial logit model. RA estimators model the potential outcome of treatment without any assumptions about the treatment model. Augmented, inverse-probability weighted (AIPW) and inverse-probability weighted, regression adjustment (IPWRA) estimators address the outcome model

(1) $$Y_{i}=f(X_{i}\text{,}\beta )+\varepsilon_{i}\text{,}$$

as well as the treatment model,

(2) $$\mathrm{Pr}(T_{i}=1\text{,}2)=h(Z_{i}\text{;}\lambda )+\omega_{i}\text{.}$$

***X*** is a vector of covariates that influence the outcome Y and ***Z*** is a set of covariates explaining treatment assignment T, which may overlap. By exploiting inverse-probability weights, these estimators control for selection.

AIPW and IPWRA enable consistent estimation of treatment parameters when either the outcome model, the treatment model, or both are correctly specified. For this reason, both the AIPW and IPWRA are called “doubly robust.” The AIPW has been termed the “efficient influence function” (Cattaneo, 2010); the IPWRA is also known as Wooldridge’s “doubly robust” estimator (2010: 930). AIPW and IPWRA estimators can be more efficient than RA (Cattaneo, 2010, Cattaneo et al., 2013, Glynn and Quinn, 2010). A recent example of the application of the binary AIPW estimator to analyze agricultural innovations in Malawi is found in Haile et al. (2017). Esposti (2017) applied the multivalued, Cattaneo (2010) model to evaluate the impact of the 2005 reform of the Common Agricultural Policy on farm production choices using treatment intervals.

Identification of the treatment effect relies on the satisfying the properties of conditional mean independence, which stipulates that the treatment does not affect the mean of the outcome variable. The multivalued case relies on a weaker assumption than in the binary case (Esposti, 2017, Cattaneo, 2010). Among our estimators, weighting by the inverse of the estimated propensity score can achieve covariate balance and creates a sample in which the distribution of covariates is independent of treatment status (Karamba and Winters, 2015). Potential bias generated by unobservable characteristics remains. We address this requirement by introducing plot manager characteristics (gender and marital status, generation, education) into the treatment model to control for intrinsic, unobserved factors related to management and access to resources within the extended family household (EAF). We also examine the common support condition. Models are estimated in STATA 14.

### 3.3 Variables

The conceptual basis of our variety choice model is the non-separable model of the agricultural household, reflecting production by a farm family enterprise (EAF) that primarily deploys its own labor supply and land in an effort to produce staple food requirements in a situation with market imperfections. In our survey data, we find virtually no evidence of land or labor markets; farm families consume, sell, and give their harvests to others. According to this framework, we expect household capital endowments (wealth, education, labor supply) and proximity to market infrastructure to affect transactions costs and thus the likelihood of acquiring inputs in sorghum production. Although we would argue that typically, sorghum seed is not viewed as a purchased input in the same way as fertilizer or herbicides, endowments also affect access to information and knowledge about new seed types.

In Mali, access to formalized extension structures (“encadrement”) substitutes to some extent for commercial markets, influencing farmer access to inputs and services of various kinds, including subsidized fertilizer. To express “encadrement,” we include a variable measuring the share of all plot managers in the village who are members of a registered farmer cooperative. The market network extends to weekly fairs conducted in villages. We include a dummy variable for the presence of a weekly fair in the village of the EAF.

Finally, as described above, we recognize the social organization of production in this region of Mali, and add the characteristics of the plot manager (education of the manager, whether the plot is managed by an individual other than the head; whether the manager is the wife or son of the head) among our explanatory variables. We also control for physical features of the plot, including time in minutes to travel from homestead to the plot and whether any structure has been built on the plot to offset soil and water erosion. Table 1 shows the definitions and means of our independent variables in the ordered logit model.

**Table 1:** Adoption model explanatory variables, definitions and means.

Variable	Definition	Mean or %
*Plot manager characteristics*		
Individually-managed	Plot managed individually by male or female who is not the EAF head or designate = 1, else 0	27.5
Education	Plot manager attended primary school = 1, 0 else	15.5
Wife	Plot manager is senior wife of the head of EAF = 1, 0 else	14.9
Son	Plot manager is son of the head of EAF = 1, 0 else	10.9

*Plot characteristics*		
Location	Time in minutes to travel from homestead to the plot	20.2
Erosion control	Any anti-erosion structure built on plot = 1, 0 else	17.4

*Household characteristics*		
Assets	Total value of non-livestock assets (ln FCFA)	14.0
Labor supply	Number of adults 12–55 years of age/total area operated	0.967

*Market characteristics*		
Cooperative	Share of plot managers in village who are coop members	0.379
Market	Weekly market fair in village = 1, 0 else	20.9

Percent reported for binary variables. n = 728.

In estimating multivalued treatment effects, we seek to quantify the potential outcomes that express changes in the supply of sorghum to the EAF and associated changes in the consumption patterns of the EAF. Referring to equation (2), our treatment specification (*h*) is a multinomial logit with *T* equal to zero if a local variety is grown on the plot, 1 if an improved variety is grown, and 2 if hybrid seed was planted. The outcome models (*f*) in Eq. (1) are specified as linear or fractional response in form, depending on the variable of interest.

Outcome variables (*Y*), definitions and means are shown in Table 2, including yield, dietary diversity, the value share of sorghum and other cereals in the food purchases during the week preceding the survey, the share of sorghum in the total quantity of cereals consumed from the harvest, and the share of sorghum harvested that was sold. Of particular interest, the Household Dietary Diversity Score (HDDS) represents a count of the different food groups consumed during the 7 days preceding the survey (Swindale and Bilinsky, 2005). The variable “FreqHDDS”, used here, augments the score to reflect the number of times a food group is consumed (Arimond and Ruel, 2002). For each food group, the EAF receives a score of 0 for frequencies fewer than four times per week, a score of unity for frequencies from 4 to 6 (inclusive) times per week, and a score of 2 for frequencies of seven or more. With ten groups, the hypothetical range of the sum is 1–20.

**Table 2:** Outcome variables, definitions and means, by treatment.

Outcome	Definition	Local variety	Improved variety	Hybrid seed
Yield	Sorghum kgs harvested/ha (GPS)	782.4	873.9	994.6
FreqHDDS	Household Dietary Diversity Score (HDDS) with frequency of consumption; see text	11.8	11.8	12.7
Sorghum purchase share	Value share of sorghum in consumption expenditures during 7 days before survey	0.075	0.0841	0.00536
Other cereal purchase share	Value share of other cereals in consumption expenditures during 7 days before survey	0.266	0.285	0.39
Sorghum consumption share	Quantity share of sorghum in cereals consumed during crop season	0.38	0.322	0.364
Sharesold	Share of sorghum harvest sold	0.0591	0.113	0.179

n = 728.

Without controlling for other factors, average yields and mean dietary diversity appear to increase with the improvement status of sorghum varieties. The mean share of sorghum in recent food purchases declines with improvement status, while the mean value share of other cereals in recent food purchases rises. The quantity share of sorghum in cereals consumed during the last cropping season is lower, and the share sold of the previous season’s harvest increases, when improved varieties or hybrids are grown.

For yield, we specify a model where *Y* is sorghum yield in kg/ha and ***X*** is a vector of agricultural inputs applied on sorghum plots, including quantities per ha (seed, adult male labor, adult female labor, children’s labor, fertilizers), as well as physical plot characteristics. ***Z*** is a vector of the same plot manager covariates that are included in the adoption analysis (with the exception of individual management; regressions did not converge with this covariate). Definitions and means of control variables are shown in Table 3.

**Table 3:** Effect model control variables, definitions and means.

Variable	Definition	Mean or %
*Production inputs*		
Seed	Quantity of seed (kgs)	12.4
Fertilizer	Quantity of fertilizer applied (kgs)	29.3
Male labor	Number of adult male person-days (14 years and above)	90.5
Female labor	Number of adults female person-days (14 years and above)	30.7
Child labor	Number of children person-days (under 14 years)	14.4

*Plot characteristics*		
Location	Time in minutes to travel from homestead to the plot	20.2
Erosion control	Any anti-erosion structure built on plot = 1, 0 else	17.4

*Plot manager characteristics*		
Education	Plot manager attended primary school = 1, 0 else	15.5
Wife	Plot manager is wife of the head of EAF = 1, 0 else	14.9
Son	Plot manager is son of the head of EAF = 1, 0 else	10.9

*Consumption factors*		
Market	Weekly market fair in village = 1, 0 else	20.9
Household size	Number of EAF members	15.5
Transfers	Income from absent members in previous 12 mos (FCFA)	71,868
Assets	Value of EAF non-livestock assets (ln FCFA)	14.0

Percent reported for binary variables. n = 728.

For consumption outcomes, following the conceptual basis of the agricultural household, we consider that relevant factors include both the supply side and those that affect outcomes through a constraint on expenditures. The specification includes the production function inputs of the yield model (the supply outcome) and plot manager characteristics as treatment covariates, adding the covariates that are likely to condition consumption, given the amount produced as outcome covariates. These include household size, transfer receipts received in the year preceding the survey from absent household members (exogenous income), as well as household wealth in assets and the presence of a weekly market fair in the village, which affect transactions costs of purchasing consumption goods.

## 4 Findings

### 4.1 Ordered logit regression

The ordered logit regression model explaining the adoption of sorghum varieties by improvement status is shown in Table 4^2^. Marginal effects of this model are shown in Annex Table 1.

**Table 4:** Ordered logit model explaining adoption of improved sorghum.

	Improvement status
Individually-managed	−0.573*
	(0.327)
Wife	0.882**
	(0.344)
Son	0.407*
	(0.240)
Education	0.878***
	(0.204)
Location	0.00207
	(0.00363)
Erosion control	−0.475**
	(0.204)
Assets	0.206***
	(0.0785)
Labor supply	0.191**
	(0.0826)
Cooperative	−0.0147
	(0.353)
Market	−0.154
	(0.197)
Constant cut1	3.605***
	(1.143)
Constant cut2	6.049***
	(1.148)
Observations	728

Robust standard errors in parentheses. n = 728.

Improvement status: 0 = local variety; 1 = improved variety; 2 = hybrid seed.

*** p < .01.

** p < .05.

* p < .1.

Plot manager characteristics, which reflect the social organization of farming in this region of Mali, are key determinants of variety adoption in sorghum production. These features are not often included in adoption studies, which usually focus on household characteristics. Individual management of a plot, compared to collective management of a plot by the EAF head, generally reduces the likelihood that improved varieties of sorghum or hybrid seed are grown. Thus, potentially higher-yielding sorghum seed is allocated in first priority to the collective plots of the extended family, which are destined first and foremost to their collective needs. However, management by the senior wife of the head increasing the chances that improved varieties, and especially hybrids, are grown in the plot. The effect of management by the son is also positive and significant but weaker in magnitude and significance. These results reflect their status relative to other plot managers, such as other wives, daughters-in-law, brothers and fathers of the head.

Clearly, improved seed is distributed among plot managers, although some members appear to have better access within the household. Seed is an input that is neutral to scale when other complementary inputs such as fertilizer and irrigation are not used. Sorghum is not heavily fertilized in Mali and all of the plots surveyed were rainfed. Culturally, the right to seed is still an important social norm, so that constraints on overall access are not expected to be binding unless a seed variety is newly introduced. In addition, sorghum is a food staple, and qualitative work in this region has underscored the fact that women are increasingly planting sorghum on their individual fields, often grown as an intercrop with other crops they grew previously (such as legumes), in order to supplement the family food supply. As part of the current outreach strategy of the national sorghum improvement program, women farmers, in addition to men in the household, have been approached in recognition of their roles in sorghum production.

Attainment of primary education by the plot manager is strongly significant for adoption of improved varieties, and even more so for sorghum hybrids. Despite that variety information is transmitted by word of mouth, in general, primary education broadens interest in and access to information and services, supporting innovation. We expect the ability to read and write strongly affects receptiveness and access to new information, techniques, or technologies.

While plot location does not influence the likelihood of growing improved seed, erosion control structures on the plot (stone contour walls, contour bunds, living fences) are negatively associated with improvement status. Thériault et al. (2016) reported similar findings in Burkina Faso. Presence of anti-erosion structures on plots typically reflects the slope of the land and dissemination efforts by formal cooperative structures more than variety type. Hybrids have been more recently introduced; the average time since initial construction of stone bunds on sorghum plots in the sample is 10 years. Furthermore, while women managers in our sample grow hybrids, they are less likely to have anti-erosion structures on their smaller plots.

As in the broad adoption literature (Feder et al., 1985, Foster and Rosenzweig, 2010), capital endowments (household wealth and household labor supply) are strongly significant in predicting the use of improved sorghum varieties. On the other hand, neither the extent of membership of village plot managers in a registered cooperative nor the presence of a weekly market fair in the village appears to influence the likelihood that improved varieties of sorghum are planted on a plot. The explanation for the first result is that registered cooperatives are primarily conduits for inputs and services related to cotton production, which also includes maize seed but not sorghum seed. Fertilizer subsidies, while facilitated by cooperatives, have also been facilitated by other associations and are in principle available to sorghum growers, though at a lower rate (Thériault et al., 2015). Improved sorghum seed has been introduced occasionally by external organizations and programs, but directly and indirectly via farmers’ associations. However, diffusion has occurred primarily from farmer to farmer, among those who are members of farmers’ associations, but not exclusively. Concerning the local market, it is still the case that little of the sorghum seed planted by farmers in this region passes through commercial markets or agrodealers, despite efforts by donors to stimulate the development of seed markets (Haggblade et al., 2015).

### 4.2 Multivalued treatment effects

Estimates of average treatment effects (ATEs) are shown for all outcomes and the three estimators in Table 5. In terms of significance of effects, results are generally, but not always consistent across models. The most noteworthy differences in both significance and coefficient magnitudes tend are between the regression adjustment approach (the baseline model), and the other two approaches, which explain both treatment assignment and outcome. If the overlap assumption holds, both the AIPW and IPWRA estimators have the “double robust” property. The density distributions of the estimated probabilities for the three groups, shown in Annex B, show little mass around zero or one, supporting the overlap assumption.

**Table 5:** Average treatment effects, adoption of improved and hybrid sorghum.

		RA	AIPW	IPWRA
		coef	coef	coef
Yield	ATE			
	Improved	203.8	173.0	204.3
	Hybrid	478.5**	779.4**	1054.8***
	ATE %			
	Improved	0.3357052	0.2275826	0.3486967
	Hybrid	0.7880124**	1.025119**	1.8005***

Sorghum purchase share	ATE			
	Improved	0.000373	−0.00828	−0.00725
	Hybrid	−0.0679***	−0.0694**	−0.0699***
	ATE %			
	Improved	0.0053511	−0.1082744	−0.0946649
	Hybrid	−0.9744761***	−0.9076069**	−0.9125638***

Sorghum quantity share	ATE			
	Improved	−0.0671***	−0.0729**	−0.0716***
	Hybrid	0.0043	−0.00137	−0.00331
	ATE %			
	Improved	−0.1126063	−0.1902753**	−0.1867071**
	Hybrid	0.0373155	−0.0074596	−0.0135241

Sharesold	ATE			
	Improved	0.0588**	0.0816**	0.0781**
	Hybrid	0.1735***	0.1090***	0.1106***
	ATE %			
	Improved	0.937192**	1.242769**	1.166684**
	Hybrid	2.556126***	1.601737**	1.614431**

Other cereal purchase share	ATE			
	Improved	0.0355	0.0649*	0.0591*
	Hybrid	0.0301	0.0513	0.0629
	ATE %			
	Improved	0.1835128	0.2393542*	0.2194374*
	Hybrid	0.0811734	0.2009184	0.2316561

FreqHDDS	ATE			
	Improved	0.388	−0.0915	−0.0873
	Hybrid	1.207	0.841**	0.903**
	ATE %			
	Improved	0.0369894	−0.0077307	−0.0073753
	Hybrid	0.1150511	0.0710718**	0.0762431**

n = 728.

* p < .10.

** p < .05.

*** <.001.

Yield effects are strongly significant and of a large relative magnitude for sorghum hybrids, but not for improved varieties, relative to local varieties. Yield advantages are between 479 and 1055 kg/ha, depending on the model, which represents 79–180% of the untreated mean of the local varieties grown by farmers (Table 5). This result confirms findings reported by Rattunde et al. (2013), which were generated by on-farm trials. The fact that the yield advantages of 34–35% estimated for improved varieties are not statistically significant probably reflects the underlying variability of yields under farmers’ conditions for this heterogeneous category of materials that combines older and more recent introductions.

Meanwhile, the expenditure share of sorghum is reduced by growing hybrids, as measured during the week preceding the visit when enumerators asked for consumption information. Since interviews occurred three to four months after the harvest, these do not represent either a post-harvest pattern or “hungry” season pattern.

At the same time, the effect of growing improved varieties on the share of sorghum consumed from the harvest is negative and statistically significant. As yields rise, the share needed to satisfy consumption needs declines given no substantial changes in household size. This result could also be explained by the fact that higher sorghum yields can enable farmers to release land for the production of other cereals that they would like to consume. Among these villages, for example, maize is both grown and consumed more than was the case in the past.

Notably, the share of the sorghum harvest sold rose by large percentages (about 160% of the untreated mean) when improved seed was grown, and even more so for hybrid seed. This finding suggests that growing improved sorghum varieties or hybrids could contribute to commercializing a food crop for which no formally developed market channel has been developed. Assuming a consumer price of 150 FCFA in the hungry season, and 90 FCFA as the sales price just after harvest, improved varieties and hybrids (combined) give the farmers the potential to increase their sales revenue by between 4644 and 7740 FCFA and 6500–10,836 FCFA depending on model.

Earnings from additional sales might be utilized to purchase other cereals or other food items. Consistent with this point, the effect of growing improved varieties is positive on the expenditure share of other cereals. Neither this nor the effect on sorghum consumption shares is apparent for hybrids, but small areas were planted to hybrid seed per household and despite higher per ha yields, the increment to total amounts harvested by the household may not have been large. When the frequency of consumption is taken into account, growing hybrids does appear to be significantly associated with dietary diversity in the households of hybrid growers, generating a 7–8% increase in the score in the AIPW and IPWRA models.

## 5 Conclusions

In this analysis, we have contributed to a relatively sparse literature on the adoption and impacts of improved sorghum varieties in the Sudan Savanna of West Africa, including the first sorghum hybrids released in Sub-Saharan Africa that were bred largely from local germplasm. The analysis is based on primary data collected in Mali, where the sorghum hybrids were developed through participatory, on-farm testing, and released as a pilot initiative by the national program.

We apply two econometric approaches that are infrequently used in the seed adoption literature, in order to differentiate improved varieties from hybrids. To identify the determinants of adoption, we applied an ordered logit model to reflect the differences in the attributes of the germplasm and the length of awareness and diffusion periods for improved as compared to hybrid seed in our study area. Reflecting the social organization of sorghum production in Sudan Savanna, which involves production by extended families on multiple plots managed by different household members, we tested the significance of plot manager characteristics, as well as the plot, household and market characteristics in our regressions. Status within these households, and access to inputs and other farm resources, is conferred to some extent by gender (combined with marital status), and generation (age or seniority). We also include education among these factors.

Then, we applied a multivalued treatment effect approach to evaluate the differential effects of adoption of sorghum varieties and sorghum hybrids on extended families. In terms of outcomes, we evaluated both supply outcomes (yield per ha at the plot level) and consumption outcomes (dietary diversity, share of sorghum and other cereals in quantities consumed and purchased, and sales). We applied three statistical models, including a baseline regression adjustment and two “doubly robust” approaches that model both treatment and outcomes, controlling for selection. We used plot manager characteristics (gender, generation, education) to capture intrinsic, unobserved heterogeneity, while controlling for physical characteristics of the plot, household and market covariates.

Compared with other adoption studies, we find statistical significance of plot manager characteristics related to intrahousehold status. The improvement status of the sorghum grown is positively associated with collective plot management by the head. This finding suggests that heads prioritize the use of seed of new, potentially higher yielding varieties on fields destined to meet the starchy staple needs of the extended family as a whole. However, among plots managed by household members other than the head, the senior wives and sons of heads or more likely also to be growing improved seed or hybrids. Attainment of primary education by the plot manager is also a significant determinant, consistent with the notion that formal education is correlated with various information pathways that can affect access to improved varieties and hybrid seed.

As expected in the broad adoption literature, household capital endowments are significant determinants of adoption. Contrary to common findings, being a member of a formal cooperative has no effect on adoption of improved sorghum varieties because these primarily facilitate access to fertilizer and other credit to cotton growers. Further, the fact that the presence of a weekly market fair in the farmer’s village has no effect on improvement status is not unexpected. The commercial market for seed remains underdeveloped, and most improved seed and sorghum hybrids have been initially introduced through programs. Subsequently, improved seed has typically diffused from farmer to farmer through customary channels.

The multivalued treatment effects model shows that yield effects are strongly significant and of a large relative magnitude for sorghum hybrids, but not so much for improved varieties, relative to local varieties. Growing sorghum hybrids also reduces the share of sorghum in food expenditures a few months after harvest. Greater production from growing improved varieties reduces the share of sorghum consumed from the harvest. The share of the sorghum harvested that is sold rises when improved varieties and sorghum hybrids are grown. Meanwhile, the impact of growing improved varieties is positive on the share of other cereals in consumption expenditures. Growing sorghum hybrids also has a meaningful and significant effect on household dietary diversity, reflecting the capacity of household to plant or purchase other food items when yields rise. On the basis of these findings, we conclude that adoption has the potential to contribute to diversification of consumption patterns and to greater commercialization by smallholders.

## 6 Implications for policy and future research

Generated with household survey data, our findings regarding the yield advantages of sorghum hybrids support previously published, on-farm research by Rattunde et al. (2013). We and Rattunde et al. (2013) demonstrate the potential importance of heterosis for increasing grain yield under farmers’ field conditions—which is of significance for agricultural research policy and for farmers elsewhere in West Africa. Results have clear consequences for sorghum improvement strategies. Improving local germplasm is a useful approach, including heterosis. Achievements reflect that farmers were involved in the testing of hybrids which are both higher-yielding and considered to be acceptable for local use. Beyond productivity effects, the analysis also lends insight into the potential for improved varieties, and particularly, locally-developed sorghum hybrids, to contribute to dietary diversification and commercialization of a staple food crop. Like other starchy staples in the region, sorghum has not benefited nearly as much as many cash crops from investments in marketing programs and related infrastructure.

Indeed, ready access to a local market and access to formal cooperative structures do not yet seem to play a role in the use of improved seed or sorghum hybrids in the Sudan Savanna of Mali. Given that sorghum is a food crop grown primarily by poor, dispersed smallholders in semi-arid areas, policy makers in West Africa must be realistic in their expectations of the extent and form of private sector interest in supplying sorghum seed. In Mali, parapublic models, and decentralized means of seed supply to more remote areas through farmer seed producer associations appears to be a workable model in regions where the formal cooperative structures are inactive (Coulibaly et al., 2014, Haggblade et al., 2015). Seed dissemination strategies should be integreated into locally acceptable norms and patterns of local seed distribution, inlcuidng sales by locally trusted farmers. We are not aware of greater private sector engagement in neighboring Burkina Faso or Niger.

In our analysis, we incorporate the social organization of production by controlling for plot management type (collectively- or individually managed) and manager characteristics (gender, generation, education). However, we have not depicted the process of intrahousehold decision-making about seed use—a potential area of future research. In our study area, anecdotal evidence suggests that women are increasingly growing sorghum on the individual plots they manage to address the nutritional needs of their children and meet the costs of clothing, school, and health care. Where this proves to be the case, any policy aiming to promote the development and diffusion of improved varieties of sorghum (especially hybrids) should recognize the potential role of women members of the farm family enterprise in planting and producing sorghum. This pattern is clearly a change in cultural norms; the conventional wisdom has been that women were not involved in sorghum production outside the collective fields of the family. To encourage higher adoption rates, our results indicate that channels of introduction for seed should incorporate not only the household head but also all economically active members of the EAF. The role of senior women in the EAF is seen to be strong in our sample, which reflects a combination of factors, including that the national sorghum program in Mali has made an effort to ensure that women contribute and benefit from variety testing programs and related activities.

Concerning these substantive issues, testing findings in other regions of West Africa where improved varieties and sorghum hybrids have been introduced over time will be important for the formulation of national and regional policy.

## Figures and Tables

**Table:** 

		Delta-method		
	dy/dx	Std. Err.	z	P > z
Individually-managed plot				
Local variety	0.13004	0.073798	1.76	0.078
Improved variety	−0.09757	0.055008	−1.77	0.076
Hybrid variety	−0.03247	0.019407	−1.67	0.094

Wife				
Local variety	−0.20016	0.077019	−2.6	0.009
Improved variety	0.150182	0.057427	2.62	0.009
Hybrid variety	0.049976	0.020973	2.38	0.017

Son				
Local variety	−0.09246	0.05413	−1.71	0.088
Improved variety	0.069376	0.040743	1.7	0.089
Hybrid variety	0.023087	0.013827	1.67	0.095

Education				
Local variety	−0.19928	0.044414	−4.49	0.000
Improved variety	0.149523	0.033556	4.46	0.000
Hybrid variety	0.049757	0.013174	3.78	0.000

Anti-erosion				
Local variety	0.107774	0.045797	2.35	0.019
Improved variety	−0.08086	0.033982	−2.38	0.017
Hybrid variety	−0.02691	0.012477	−2.16	0.031

Assets				
Local variety	−0.04677	0.017592	−2.66	0.008
Improved variety	0.035094	0.013091	2.68	0.007
Hybrid variety	0.011678	0.004828	2.42	0.016

Labor supply				
Local variety	−0.04325	0.018644	−2.32	0.02
Improved variety	0.032448	0.014146	2.29	0.022
Hybrid variety	0.010798	0.004777	2.26	0.024

^∗^Only statistically significant variables are included. n = 728.

**Figure:**
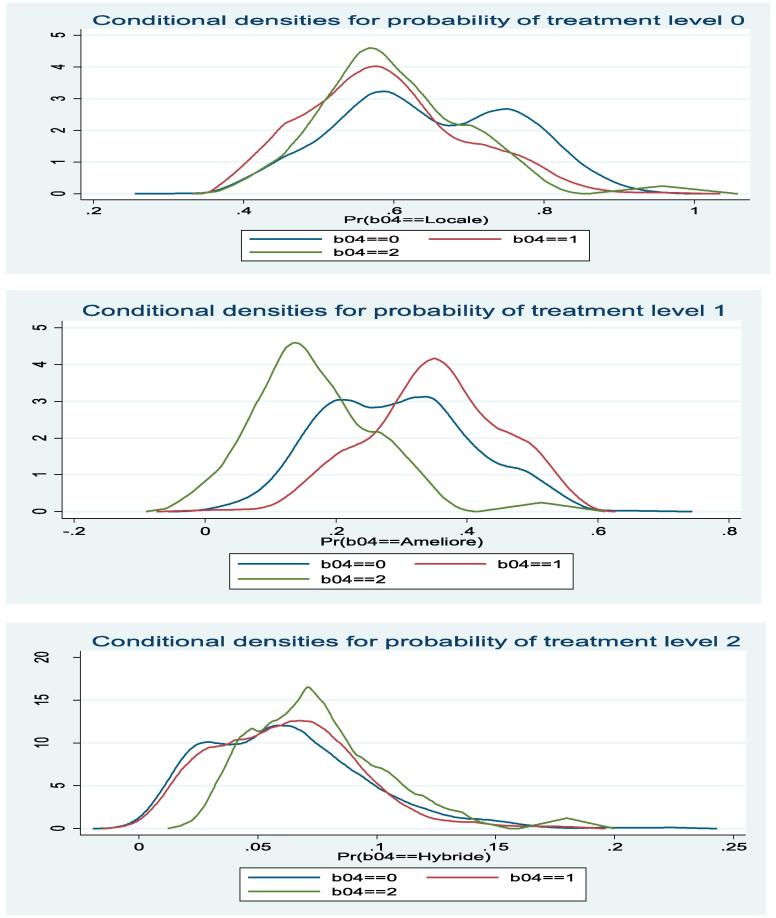


## Footnotes

2 Results of the multinomial model, which are similar in terms of significant factors, are available from the authors.
